# Evaluating farmers’ adaptation strategies to climate change: A case study of Kaou local government area, Tahoua State, Niger Republic

**DOI:** 10.4102/jamba.v8i3.241

**Published:** 2016-03-31

**Authors:** A. Moussa Tabbo, Zakou Amadou, Agada B. Danbaky

**Affiliations:** 1 Department of Agricultural Economics, Usnamu Dan Fodiyo University, Nigeria; 2 Department of Rural Sociology and Economics, Tahoua University, Niger Republic; 3 Department of Economics and Management, Tahoua University, Niger Republic

## Abstract

The study discusses local farmers’ strategies of coping with and building resilience against the negative impact of climate change. Information for the discussion was from data collected using a set of structured questionnaires from interviews scheduled with 128 farmers. The questionnaire was based on previous literature and direct reconnaissance interview with farmers, which culminated in 13 strategies used for the study being reported. For each question, respondents were asked to choose their best and worst strategies. Thus, the difference between the best and worst strategies consistent with random utility theory has been used for the modelling. Results show that semi-transhumance, various handicrafts making, rural migration, small-scale vegetable production and small-scale river exploitation were the most important strategies identified, whilst water transport and vending, shifting cultivation, gypsum mining, gathering and trading of wild fruits and edible plants as well as cattle and sheep fattening were the least appreciated strategies identified amongst the farmers facing climate change. These findings are therefore imperative for planning farmers’ capacity-building and resilience against climate change projects to ensure sustainability in the study area.

## Introduction

The climate change defined as sustainable and large-scale modification of the climate is considered as one of the biggest threats to our planet. Human actions change the climate at the local and regional level, whilst use of fossil energy influences the climate at the global level.

According to Niasse, Afouda and Amani (2004), Africa is one of the continents that are the most vulnerable in facing the negative effects of climate change. Thus, the economies of African countries are mainly dependent on natural resources, which are sensitive to climate; they are not able to develop coping strategies against climate change (OECD 2006). Moreover, their agricultural productions based on backward techniques fail to meet the needs of an increasing population, thereby creating a real threat to food security.

Previous studies conducted in Niger show that the climate change not only affects the agricultural production, the food security, the health and the biodiversity, but also accelerates desertification. To solve these problems, several measures such as adaptation to certain impacts (decrease in agricultural output, the shortage of rainfall and lack of pasture) and the reduction of greenhouse gas emission (carbon sequestration) have been proposed. Also, others strategies such as resources trading (straws, woods, wild fruits and fish) and socio-economic activities (trade and various handicraft making) have been initiated in short term to help farmers to adapt. Meanwhile, an additional diversification of economic activities aims at compensating losses and reductions of harvests, developing improved seeds more resistant to drought and integrating water resource management, which are some guidelines proposed for intermediate- and long-term adaptation.

According to the communal development plan report (2011), the local government of Kaou fully situated between the Sahara and the Sahel zones is affected by the conjugated effects of desertification, water and wind erosions, high pressure on agricultural land and the progressive decrease in the amount of rainfall. This has resulted in consequences such as natural resource degradation, decrease in agricultural production and shift in agricultural line explaining by the search of capacity development to enhance resilience through the multiplication of strategies. The application of effective change in the climate adaptation strategies will improve resilience of hundreds of millions of people living in developing countries. Thus, involving local authorities and community-based organisations in the development of adaptation strategies will be important.

That is why projects, nongovernmental organisations and development partners have financially and technically supported the local government authority to develop household adaptation strategies so as to reinforce their resilience against the negative impacts of climate change. However, the evaluation of farmers’ adaptation strategies against climate change has not been undertaken in the study area where most projects have been introduced thereby given little attention to farmers’ views.

The present study will contribute to address weaknesses observed in project planning and execution. The main objective of the research is to determine and evaluate farmers’ adaptation strategies against climate change. Specific objectives are to determine the relative importance of each farmer’s strategy and to draw a general tendency about the most appreciated strategies.

### Conceptual framework and background

We suppose that farmers maximise their utility when they decide to choose one strategy as the best and another as the worst to adapt against the negative effect of climate change. Therefore, nongovernmental organisations, the government and development partners should consider what strategies are the most important to farmers in order to enhance their resilience against climate change. In addition, the understanding and the determination of the most important strategies allows farmers to elaborate economically feasible and environmentally friendly projects of development, which is compatible with Australian researcher philosophy stating that people do the project and the project does the development.

The application of best and worst scaling (BWS) has been widely used to study consumers’ behaviour because it is consistent with random utility theory. The BWS method was first developed by Jordan Louvriere, whilst he was working at the University of Alberta in 1987. He stated that this BWS method is better than other methods such as category rating and ranking scales because it not only forces respondents to discriminate amongst items but also helps them to compare both intra- and inter attributes. Flynn *et al.* (2007) also reported that results obtained from BWS can be easily interpreted. Based on this approach, a set of strategies is presented to farmers and they are asked to choose the best and worst strategies. The BWS method has recently been used to determine preferences for sustainable farming practices (Sackett, Shupp & Tonsor 2013), the relative importance of food values to consumers (Lister *et al.* 2014; Lusk & Briggeman 2009), consumers’ preferences for quality and safety attributes of milk products (Amadou 2014), preferences for US Department of Agriculture market reports (Ross Pruitt *et al.* 2014), preferences for which livestock production methods matter most to consumers (Brooks & Brenna 2014) and seed attribute preferences amongst resource-poor farmers in northern Nigeria (Abubakar, Amadou & Daniel 2014).

Results from recent studies show that general food values as suggested by previous research generally hold when applied to specific animal food values. Choices made by decision-makers can be modelled in a variety of ways, but mixed logit capable of handling any behavioural model is widely used (Revelt & Kenneth 1998). Therefore, choices made by farmers helping to maximise utility by choosing a set of strategies can be modelled by the difference between the best and worst strategies observed in each question. Thus, choice made by each farmer can be mathematically expressed:

$$U_{ij}=V_{ij}+\varepsilon_{ij}$$ [Eqn 1]

where *U_ij_* is the utility of a farmer, *i* choosing a given strategy *j, V_ij_* and *ε_ij_* are, respectively, the deterministic and stochastic components of utility.

### Data collection method

The BWS method helps to determine the best strategies and the worst strategies and has been used by farmers to cope against the negative effects of climate change. Thus, the balanced incomplete block design was used to build questionnaires that served as a tool for data collection. This block design is balanced with respect to rows and each strategy is equally replicated through the questionnaire so as to maintain the likelihood principle. Based on previous studies and interviews with farmers, 13 strategies were compiled and used in the study. The R statistical software was used to generate the 13 blocks or questions having four strategies randomly assigned to each. The questionnaire having in total 13 strategies was used to collect data from farmers randomly selected. For each question, farmers were asked to choose their best and their worst strategies.

We conducted the survey in the rural county of Kaou, which was identified as the most vulnerable county in Tahoua State (CNEDD 2009). The survey is voluntary, and farmers were randomly selected to participate. In addition, four villages (Kao, Darha, Kaou and Indamane) were randomly selected and only one person of specific gender in a given household was interviewed to increase the diversity and the representativeness of the sample size. In total, 128 farmers were randomly selected and interviewed using face-to-face interview. A sample based on BWS is presented in Table 1.

**TABLE 1:** A sample of the best and worst scaling method.

The best strategy	Characteristics	The worst strategy
□	Shifting cultivation	□
□	Rural migration	□
□	Semi-transhumance	□
□	Brick making	□

Note: Question asked to choose the best and worst strategies you will use to adapt against climate change.

## Procedures and methods

Following the method of Lusk, in a set of *k* elements, there are *k*(*k*−1) possible combinations. The choice of a pair of strategies in the *k*(*k*−1) combinations corresponds to a maximum allocation of the choice difference. We assume that α_*k*_ represents the location of value *k* on a specific scale of importance. Thus, the level of importance for each individual person *i* can be expressed as follows:

$$P_{ik}=\alpha_{k}+\varepsilon_{ik}$$ [Eqn 2]

where ε_*ik*_ is a random term included to take into account unobserved factors.

The probability that *k* and *j* will be selected in a set as best and worst is equal to the probability that the difference between *P_ik_* and *P_ij_* will be greater than all other *k*(*k*−1) − 1 options in the choice set. Assuming that the error term has an independent and identical distribution, a multinomial logit model can be used to model the probability which is expressed as follows:

$$Prob(k\;is\;chosen\;best\;and\;j\;worst)=\frac{\exp(\beta_{k}-\beta_{j})}{\sum_{l=1}^{k}\sum_{m=1}^{k}\exp\left[(\beta_{l}-\beta_{m})-k\right]}$$ [Eqn 3]

This regression analysis was used to determine the relationship amongst several pairs of variables and to identify how a change in one affects the other. The estimation of the model helps to determine which attribute is the most preferred and which is the least preferred. Results from the estimated mixed logit model were used to rank preference. Thus, preference share was calculated based on the following equation:

$$Prob(Strategy\;j\;is\;chosen)=\frac{e^{X\beta_{j}}}{\sum_{k=1}^{j}e^{X\beta_{k}}}$$ [Eqn 4]

where *V_j_ = X*β*_j_* is the utility of the strategy *j*, whilst *V_k_ = X*β_*k*_ is the utility of the strategy *k*.

Finally, standard normal distribution has been used to determine the percentage of farmers having positive and negative preference for a given strategy. Thus, the relation between the mean and standard deviation (following the normal distribution) for each strategy has served to determine the probabilities above and below the mean.

## Results and discussions

This section summarises the results and interpretation of data analysis. For easy interpretation of results obtained from Table 3, the estimated parameters have been used to develop Tables 4 and 5.

Table 2 provides summary statistics of the respondents included in our analysis. Results show that the respondents have an average age of 41 years. Most of the respondents were men (97%), married (59%), educated (68%) and had a large family (87%) and a significant part of their revenue was spent on food (59%). Table 2 also reveals that most of the respondents ate less than three meals per day (60%), were vulnerable to climate change (95%) and practiced various handicrafts making as dominant activities (54%).

**TABLE 2:** Characteristics of survey respondents.

Variables	Description	Mean	Standard deviation
Age	Age in years	41.71	9.69
Gender	1 if male, 0 if female	0.97	0.174
Marital status	1 if married, 0 otherwise	0.59	0.495
Household size	1 if large family, 0 if small family	0.87	0.331
Education	1 if educated, 0 if uneducated	0.68	0.480
Part of revenue spent on food	1 if a portion up to 50 000, 0 if less	0.59	0.495
Number of meals eaten per day	1 if three meals per day, 0 if less than three	0.40	0.495
The degree of vulnerability	1 if yes, 0 if no	0.95	0.213
The main activity	1 if handicraft, 0 if otherwise	0.54	0.520
	1 if vegetable production, 0 if otherwise	0.24	0.434

Table 3 shows parameter estimates of the mixed logit model. Coefficients with positive sign are considered as the best strategy, whilst coefficients with negative sign are considered as the worst strategy. Results reveal that semi-transhumance, various handicrafts’ making, rural migration, small-scale vegetable production and small-scale river exploitation are positive and statistically significant, implying that these strategies are important to farmers. However, water transport and vending, shifting cultivation, gypsum mining, sheep and cattle fattening, harvesting and trading of wild fruits and edible plants and farm work are negative and statistically significant, implying that these strategies are least preferred by farmers. Table 3 also shows that the standard deviation of rural migration, shifting cultivation, farm work, straw trading, small-scale vegetable production, semi-transhumance, various handicrafts’ making, sheep and cattle fattening, gypsum mining, small-scale river exploitation and water transport and vending are statistically significant, revealing that these strategies are random in the survey population.

**TABLE 3:** The multinomial mixed logit estimates based on farmers’ adaptation strategies against climate change.

Farmers’ strategies	Parameters	Coefficients	Standard error
Rural migration	Mean of coefficient	0.8372*	0.1754
	Standard deviation	2.0102*	0.3457
Shifting cultivation	Mean of coefficient	−1.124*	0.2137
	Standard deviation	1.1506*	0.3411
Farm work	Mean of coefficient	−0.466*	0.219
	Standard deviation	2.9468†	0.4229
Brick making	Mean of coefficient	0.439	0
	Standard deviation	0.1177	0.9691
Harvesting and trading of edible wild fruits and plants	Mean of coefficient	−0.5293*	0.1591
	Standard deviation	0.2027	0.7674
Straw trading	Mean of coefficient	0.0945	0.1674
	Standard deviation	1.7912*	0.3426
Small-scale vegetable production	Mean of coefficient	0.6663*	0.1733
	Standard deviation	2.0581*	0.3491
Semi-transhumance	Mean of coefficient	1.3271*	0.1801
	Standard deviation	1.8917*	0.3083
Various handicrafts’ making	Mean of coefficient	1.2773*	0.1758
	Standard deviation	1.2484*	0.317
Sheep and cattle fattening	Mean of coefficient	−0.6702*	0.1704
	Standard deviation	1.0743*	0.3026
Gypsum’s mining	Mean of coefficient	−0.6795*	0.1784
	Standard deviation	1.4276*	0.3003
Small-scale river exploitation	Mean of coefficient	0.7298*	0.189
	Standard deviation	2.7138*	0.3735
Water transport and vending	Mean of coefficient	−1.9023*	0.3962
	Standard deviation	4.3160*	0.7076
The log-likelihood at convergence	−3729		
The log-likelihood at zero	−4134.9		
*N*	128		
Pseudo *R*^2^	0.10		

* Statistically significant at the 10% level.

† Statistically significant at the 5% level.

Table 4 provides results on the relative importance of the farmers’ adaptation strategies used in the study. The importance or weight of each strategy is computed relative to that of brick making. Results show that semi-transhumance and various handicrafts’ making are 0.8881 times more important than brick making, whilst brick making is two times more important than water transport and vending. Table 4 shows that semi-transhumance (19.79%) followed by various handicrafts making (18.83%), rural migration (12.13%), small-scale water body exploitation (10.89%) and small-scale vegetable production (10.22%) are the most preferred strategies. However, water transport and vending (0.78%), shifting cultivation (1.71%), gypsum mining (2.66%), sheep and cattle fattening (2.69%) and harvesting and the trading of edible wild fruits and plants (3.09%) are the least preferred strategies.

**TABLE 4:** The relative importance of each strategy compared with brick making.

Local farmers’ strategies	Coefficients	Relative to brick making	%
Semi-transhumance	1.3271	0.8881	19.79
Various handicrafts making	1.2773	0.8383	18.83
Rural migration	0.8372	0.3982	12.13
Small-scale river exploitation	0.7298	0.2908	10.89
Small-scale vegetable production	0.6663	0.2273	10.22
Brick making	0.439	0	8.14
Straw trading	0.0945	−0.3445	5.77
Farm work	−0.4661	−0.9051	3.29
Harvesting and trading of edible wild fruits and plants	−0.5293	−0.9683	3.09
Sheep and cattle fattening	−0.6702	−1.1092	2.69
Gypsum mining	−0.6795	−1.1185	2.66
Shifting cultivation	−1.1241	−1.5631	1.71
Water transport and vending	−1.9023	−2.3413	0.78

Table 5 reports the percentage above and below the mean for each strategy. The mean and the standard deviation presented in Table 3 have been used to determine the preference share for each strategy. Results indicate that various handicrafts’ making is preferred by 85% of farmers, against 76% for semi-transhumance, against 66% for rural migration, against 63% for small-scale vegetable production and against 61% for small-scale river exploitation. However, the strategies such as harvesting and trading of edible wild fruits and plants is avoided by 100% of farmers, against 84% for shifting cultivation, against 73% for sheep and cattle fattening, against 68% for gypsum mining and against 67% for water vending. These results show that most farmers prefer various handicrafts’ making, semi-transhumance, rural migration, small-scale river exploitation and small-scale vegetable production as their best strategies to better adapt to the shock of climate change. However, strategies such as harvesting and trading of edible wild fruits and plants, shifting cultivation, sheep and cattle fattening, gypsum mining and water transport and vending are the least preferred to farmers.

**TABLE 5:** The probability above and below the mean for each strategy.

Local farmers’ strategies	Coefficients	% above zero	% below zero
Semi-transhumance	1.327	0.76	0.24
Various handicrafts’ making	1.277	0.85	0.15
Rural migration	0.837	0.66	0.34
Small-scale river exploitation	0.73	0.61	0.39
Small-scale vegetable production	0.666	0.63	0.37
Brick making	0.439	1	0
Straw trading	0.095	0.52	0.48
Farm work	−0.466	0.44	0.56
Harvesting and trading of edible wild fruits and plants	−0.529	0	1
Sheep and cattle fattening	−0.67	0.27	0.73
Gypsum mining	−0.68	0.32	0.68
Shifting cultivation	−1.124	0.16	0.84
Water transport and vending	−1.902	0.33	0.67

## Conclusion and implications

Several strategies have been developed to help farmers to build resilience capacity against the negative effect of climate change. However, farmers’ strategies are often ignored during the planning and the execution phase of development projects. The general objective of the study is to determine and evaluate the local strategies developed by farmers to better cope against climate change. The specific objectives are to determine the relative importance of each strategy and the proportion of farmers that are above and below the mean of each strategy. Based on previous research and interview with farmers, 13 strategies were compiled and used in the study. The balanced incomplete block design approach having four strategies randomly assigned to block was used as a tool to collect data. The BWS method based on the choice of the best and worst strategies was used as a tool for data collection, whilst the multinomial mixed logit capable of modelling any discrete choice was used to analyse the data.

Results reveal that semi-transhumance, various handicrafts’ making, rural migration, small-scale river exploitation and small-scale vegetable production are the most important strategies to farmers, whereas water transport and vending, shifting cultivation, gypsum mining, sheep and cattle fattening and harvesting and trading of edible wild fruits and plants are the least important strategies to enhance farmers’ resilience against the negative effect of climate change. Results also reveal that semi-transhumance, various handicrafts’ making, rural migration, small-scale vegetable production and small-scale river exploitation are the most preferred strategies. This implies that farmers place a significant importance on the development of these strategies in order to enhance their adaptation capacity.

The study provides important complementary information for the government, the nongovernmental organisations and the development partners so as to optimise their intervention strategies and to improve the welfare of farmers by considering their point of view before preparing and executing projects designed for them. In addition, results from the study will help the different agents intervening in the field of climate change to make beneficial and strategic decisions and thereby to alleviate the impact of the negative externality caused by the climate change. A limitation of the research was considering local adaptation strategies for only one county and it is difficult to generalise results at the global level because strategies may differ from village to village, county to county, state to state and country to country. Future direction for research is to determine and evaluate the importance of development partners’ adaptation strategies against climate change on the welfare of farmers and to compare climate change adaptation strategies across villages, counties, states and countries to better harmonise global effort and thereby effectively and efficiently enhance resilience of the most vulnerable and affected groups through sharing experiences and adaptation strategies.
